# PREVALENCE OF PALMARIS LONGUS TENDON AGENESIS IN A POPULATION OF MEDICAL STUDENTS

**DOI:** 10.1590/1413-785220263401e297925

**Published:** 2026-02-13

**Authors:** Paola Dias Tagliacozzo, Camila Cassillo, Julio Cesar Gali, Wallace Rodrigo Dantas, Leonardo Goya Machado, Edie Benedito Caetano

**Affiliations:** 1 Pontificia Universidade Catolica de Sao Paulo, Faculdade de Ciencias Medicas e da Saude, Sao Paulo, SP, Brazil.; 2 Pontificia Universidade Catolica de Sao Paulo, Faculdade de Ciencias Medicas e da Saude, Departamento de Cirurgia, Sao Paulo, SP, Brazil.

**Keywords:** Tendons, Anatomic Variation, Wrist, Anatomic Landmarks, Tendões, Variação Anatômica, Punho, Pontos de Referência Anatômicos

## Abstract

**Objective:**

To assess the prevalence of palmaris longus (PL) tendon agenesis in a population of medical students.

**Methods:**

The presence of the palmaris longus tendon was evaluated using Schaeffer's test, applied bilaterally in 100 medical students, totaling 200 evaluated antimeres. The data were analyzed for unilateral or bilateral presence of the tendon, in relation to gender, laterality (right or left), and the occurrence of agenesis.

**Results:**

Agenesis of the palmaris longus tendon was observed in 32 out of 200 evaluated antimeres (16%). Among female participants, agenesis was identified in 21 antimeres (21%), whereas in males it was observed in 11 antimeres (11%). Tendon absence was more frequent in the left upper limb, regardless of gender.

**Conclusion:**

The prevalence of palmaris longus tendon agenesis in the studied sample was 16%, with a higher frequency among females. Tendon absence was more common on the left side in both genders. ***Level of Evidence IV; Case series.***

## RESUMO

**Objetivo:**

Avaliar a prevalência da agenesia do tendão do músculo palmar longo (PL) em uma população de estudantes de medicina.

**Métodos:**

A presença do tendão do músculo palmar longo foi investigada por meio da manobra de Schaeffer, aplicada bilateralmente em 100 estudantes de medicina, totalizando 200 antímeros avaliados. Os dados obtidos foram analisados quanto à presença unilateral ou bilateral do tendão, em relação ao sexo, à lateralidade (direita ou esquerda) e à ocorrência de agenesia.

**Resultados:**

A agenesia do tendão do músculo palmar longo foi observada em 32 dos 200 antímeros avaliados (16%). Entre as mulheres, a agenesia foi identificada em 21 antímeros (21%), enquanto entre os homens, foi observada em 11 antímeros (11%). A ausência do tendão foi mais frequente no membro superior esquerdo em ambos os sexos.

**Conclusão:**

A prevalência da agenesia do tendão do músculo palmar longo na amostra estudada foi de 16%, sendo mais comum em mulheres. A ausência do tendão apresentou predomínio no lado esquerdo, independentemente do sexo. ***Nível de Evidência IV; Série de Casos***.

## INTRODUCTION

The *palmaris longus* (PL) is a superficial muscle covered by the anterior fascia of the forearm,^1–3^ being responsible for the flexion of the wrist and the tension of the palm aponeurosis.^2–5^ It originates from the medial epicondyle of the humerus, extending medially to the carpal's radial flexor muscle and laterally to the carpal's ulnar flexor, entering the palm aponeurosis.^2,3,6^ It is innervated predominantly by the median nerve and can, in some cases, receive innervation from the ulnar nerve, and is vascularized by recurrent ulnar arteries.^1,7^ This muscle is considered to be the most variable of the human body, and may present variations in duplication, agenesis, position and insertion.^8,9^ According to Yammine et al.^10^, the variations in the PL are associated with a dominant pattern of autosomal inheritance with incomplete penetration. There is evidence of its evolutionary role: in arboreal primates, the muscle is consistently present, assisting in climbing and grasping objects; whereas in bipedal or predominantly terrestrial primates, its presence becomes more variable, PL has become less necessary, which justifies its vestigial characteristic.^5,8,11,12^

The prevalence of PL tendon agenesis is approximately 15%.^2,5^ Epidemiological studies suggest that the regression of this muscle may be related to the migration of the *Homo sapiens* from the African continent, as Caucasian populations present higher rates of PL agenesis (26%) compared to descendants of Asian peoples (12%) and African peoples (6%).^7,13^ Some articles also indicate that the absence of PL occurs more frequently unilaterally, predominantly on the left side, and is more common in female sex.^2,12,14,15^

Although it is considered a vestigial muscle, the absence of which does not compromise the movements of the hand or wrist, the PL has clinical and surgical relevance.^9,16^ It is used in reconstructive procedures, such as replacement and repair of other tendons, repair of facial paralysis and reconstruction of labia defects, in addition to serving as an anatomical reference for the location of the median nerve.^3,15,17,18^ Changes in its anatomical insertion may also be associated with clinical manifestations of carpal tunnel syndrome.^11,12,19–23^

Knowledge of the prevalence of PL agenesis is essential to provide anatomical information to surgeons, allowing them to know its variations, which are useful in planning surgeries involving the use of palmar muscle as a graft or as an anatomical reference in surgical procedures. We found few studies addressing the prevalence of this tendon in the Brazilian population. Thus, the present study aimed to identify the frequency of PL muscle tendon agenesis in a population of 100 medical students.

## MATERIALS AND METHODS

**Figure 1:**
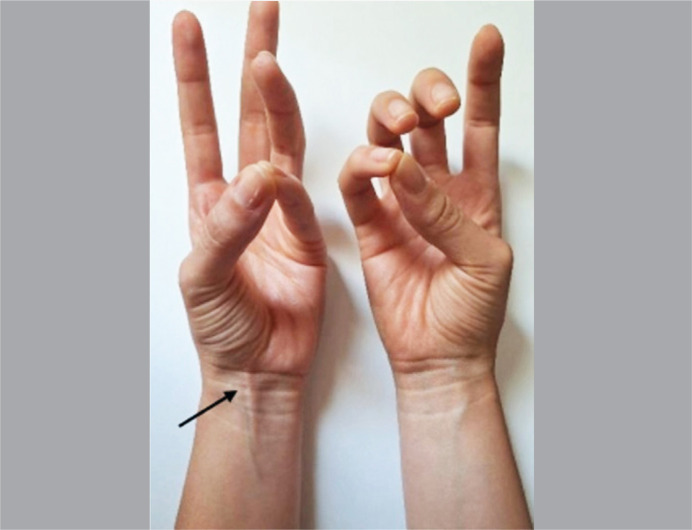
Anterior photographic view depicting bilateral performance of Schaeffer's maneuver. The PL tendon is present on the left side (arrow) and there is agenesis of this tendon on the right side.

Source: St. Archive of the Faculty of Medical and Health Sciences of the Pontifical Catholic University of São Paulo.

This research was approved by the Ethics Committee of our institution under the number 57590422.0.0000.5373. The sample was composed of 100 medical students, from the first to the sixth year of the course, aged between 18 and 30 years, of whom 50 were female and 50 were male. Only individuals without a history of trauma in the ventral area of the forearms or any history of diseases affecting the soft parts of this area were included. The selection of participants was random, depending on the availability of volunteers. Verification of the PL tendon was performed using Schaeffer's maneuver on both forearms. This test consists of asking the volunteer to keep the elbow folded at 90 degrees and perform the wrist flexion associated with the opposition of the thumb to the smallest finger (Figure 1). The presence of the tendon was considered positive when it became visible in the distal third of the wrist; if it was not possible to visualize it, its absence was recorded.

The tests for each antimere were conducted independently by two authors (PDT and CC), and the results were later compared to verify inter-observer agreement. The gender of the student and the side (or bilaterality) of the long PL tendon agenesis were recorded.

## RESULTS

Based on the data obtained, the prevalence of absence of the long PL tendon in the evaluated population was 16%. When stratified by sex, absent tendon was observed in 21% of women and 11% of men. In the female sample (n = 100 upper antimeres), the absence of the tendon was identified in 21 antimeres, with eight in the upper right limb and 13 in the left. Three volunteers presented bilateral agenesis. In the male sample (n = 100 upper antimeres), the absence was observed in 11 members, five to the right and six to the left, with the occurrence of bilateral agenesis in three individuals.

Considering the general prevalence of the absence of the PS tendon by antimeres, regardless of gender, a rate of 13% was observed in the right upper limb and 19% in the left upper limb. Stratified by sex, the data indicated that the absence on the right side was 16% in women and 10% in men; on the left side, the agenesis was 26% in women and 12% in men. The overall prevalence of bilateral PL tendon agenesis, considering both sexes, was 6% in both sexes. The results are grouped in Table 1.

**Table 1:** Prevalence of long PL tendon agenesis in the population of 100 students of the medical course, according to sex and side.

Sex	Female	Male
Unilateral and bilateral agency	21%	11%
Unilateral agenesis to the right	16%	10%
Unilateral agenesis to the left	26%	12%
Bilateral agency	6%	6%
Total of individuals	50	50

## DISCUSSION

The main finding of this study was the verification of the prevalence of PL tendon agenesis (PL) in 16% of the sample analyzed. This condition was more common among female individuals (21%), compared to male individuals (11%). Regarding laterality, the absence of tendon was more prevalent in the left upper limb (19%) than in the right (13%). Bilateral agenesis was observed in 6% of participants, with no difference between the sexes.

National studies conducted by Garcia et al.^1^ Bonsi,^24^ Pierucci et al.^25^ and Morais et al.^26^ report frequencies of agenesis of PL of 13.9%, 16.5%, 9.2% and 26.5%, in this order. The findings of this study approximate the data reported by Bonsi,^24^ whose prevalence was 16.5%, and are also comparable to the results of Garcia et al.^1^ (13.9%). On the other hand, the percentages observed by Pierucci et al.^25^ and Morais et al.^26^ showed more pronounced discrepancies. A methodological analysis of the studies of Garcia et al.^1^ and Bonsi^24^ revealed that both used the same procedure adopted in this research, i.e. Schaeffer's maneuver, and it is therefore not possible to attribute the observed divergence to the technique employed. However, Morais et al.^26^ applied four different maneuvers to confirm the absence of the PL tendon, which may justify, at least partially, the observed difference.

In comparing our results with international literature data, we found that Cohen et al.^13^ reported the absence of PL tendon in 28% of the Israeli population, while Al Risi et al.^19^ found the prevalence of agenesia in 7.5% of the Oman population. In the Pakistani population, Javaid27 observed a 12% prevalence of this tendon agenesis. On the other hand, Kapoor et al.^15^ observed the absence of tendon in 17.2% of the Indian population and Sadacharan et al.^12^ identified this absence in 12.8% of the African population. Cohen et al.^13^ suggested that PL prevalence is more strongly associated with geographical than ethnic factors, observing that individuals of different ethnicities, but residing in the same geographic region, presented similar rates of agenesis. This hypothesis may contribute to the understanding of the observed variations in relation to the data obtained in this study.

The results presented here corroborate the findings of Morais et al.^26^ Javaid,^27^ Ioannis et al.^2^ Raouf et al.^14^ and Kapoor et al.^1^ which also report greater prevalence of PL agenesis in female subjects and left antimere. These findings contrast with the data from Al Risi et al.^19^ that identified a higher prevalence of the absence of PL in males. Pierucci et al.^25^ in turn described a higher prevalence of unilateral agenesis in the left antimeric among men (15.2%) and in the right antimeric among women (18%).

Additionally, the data from this study indicate that bilateral PL agenesis occurs less frequently than unilateral agenesis, a pattern also reported by Garcia et al.^1^ Pierucci et al.^25^ Sadacharan et al.^12^ and Morais et al.^26^ No records were identified in the literature indicating a higher prevalence of bilateral agenesis of the PL tendon compared to the unilateral form.

As a limitation of our study we can cite that we did not investigate whether there was a correlation between the side of the agenesis of the PL tendon and the dominant limb and, as a clinical relevance, we can highlight that the agenesis of this tendon is present in a small portion of the population and that the bilateral absence is even smaller, which makes the PL tendon a viable option for use as a tendon graft in lesions of the finger flexors, for example, since the removal of this anatomical structure practically does not interfere with the flexion of the wrist, since other tendons, such as the ulnar flex of the carp and the radial flexor of the carp perform this function more efficiently.

## CONCLUSION

In the analyzed population, PL tendon agenesis was observed in 16% of subjects, being more common in females and left antimeres. Bilateral agenesis was identified in 6% of individuals of both sexes.

## Data Availability

The contents underlying the research are available in the manuscript.
